# Understanding the experience of initiating community-based physical activity and social support by people with serious mental illness: a systematic review using a meta-ethnographic approach

**DOI:** 10.1186/s13643-017-0596-2

**Published:** 2017-10-25

**Authors:** Helen Quirk, Helen Crank, Deborah Harrop, Emma Hock, Robert Copeland

**Affiliations:** 1 Centre for Sport and Exercise Science, Faculty of Health and Wellbeing, Sheffield Hallam University, Collegiate Hall, Collegiate Crescent, Sheffield, S10 2BP UK; 2 Centre for Health and Social Care Research, Faculty of Health and Wellbeing, Sheffield Hallam University, Sheffield, UK; 3 School of Health and Related Research, University of Sheffield, Sheffield, UK; 4 The National Centre for Sport and Exercise Medicine, Sheffield, UK

**Keywords:** Serious mental illness, Physical activity, Community, Social support, Exercise, Sport, Adults, Patient experience, Qualitative research, Meta-ethnography, Systematic review

## Abstract

**Background:**

People with long-term serious mental illness live with severe and debilitating symptoms that can negatively influence their health and quality of life, leading to outcomes such as premature mortality, morbidity and obesity. An interplay of social, behavioural, biological and psychological factors is likely to contribute to their poor physical health. Participating in regular physical activity could bring symptomatic improvements, weight loss benefits, enhanced wellbeing and when undertaken in a community-based group setting can yield additional, important social support benefits. Yet poor uptake of physical activity by people with serious mental illness is a problem. This review will systematically search, appraise and synthesise the existing evidence that has explored the experience of community-based physical activity initiation and key features of social support within these contexts by adults with schizophrenia, bipolar affective disorder, major depressive disorder or psychosis using the meta-ethnography approach. This new understanding may be key in designing more acceptable and effective community-based group PA programmes that meet patients’ need and expectations.

**Methods:**

This will be a systematic review of qualitative studies using the meta-ethnography approach. The following databases will be searched: ASSIA, CINAHL, Cochrane Central Register of Controlled Trials, EMBASE, Health Technology Assessment Database, MEDLINE, PsycINFO, Sociological Abstracts, SPORTDiscus and Web of Science. Grey literature will also be sought. Eligible studies will use qualitative methodology; involve adults (≥18 years) with schizophrenia, bipolar affective disorder, major depressive disorder or psychosis; will report community-based group physical activity; and capture the experience of physical activity initiation and key features of social support from the perspective of the participant. Study selection and assessment of quality will be performed by two reviewers. Data will be extracted by one reviewer, tabled, and checked for accuracy by the second reviewer. The meta-ethnography approach by Noblit and Hare (Meta-ethnography: synthesizing qualitative studies 11, 1988) will be used to synthesise the data.

**Discussion:**

This systematic review is expected to provide new insights into the experience of community-based group physical activity initiation for adults who have a serious mental illness to inform person-centred improvements to the management of serious mental illness through physical activity.

**Systematic review registration:**

The protocol has been registered on the International Prospective Register of Systematic Reviews (PROSPERO) on 22/03/2017; (registration number CRD42017059948).

**Electronic supplementary material:**

The online version of this article (10.1186/s13643-017-0596-2) contains supplementary material, which is available to authorized users.

## Background

### Serious mental illness

People with long-term serious mental illness (SMI) live with severe and debilitating symptoms that can negatively influence the quality of life of themselves, their families, carers and society. Common SMIs are schizophrenia, bipolar affective disorder, major depressive disorder, personality disorder, severe anxiety (including phobia and obsessive compulsive disorder), schizophreniform disorder or psychosis (including first episode psychosis). Individuals with SMI have higher levels of premature mortality and morbidity [1], and obesity is more prevalent when compared with the general population [2]. Many factors contribute to the poor physical health of people with SMI including social, biological, psychological and behavioural factors. Attempts to understand the experience of living with SMI need to consider the complex individual-level and system-level influences on behaviour [3]. Social factors include unequal provision of healthcare [4], stigmatisation and lack of social support [5]. Biological factors such as chronic disease and psychotropic medication, and psychological factors such as low self-esteem and negative thoughts can also contribute to ill-health and weight gain [6]. Smoking, poor diet and sedentary behaviour are behavioural factors contributing to the poor health of people living with SMI [6]. Modifiable behavioural risk factors such as low levels of physical activity (PA) have become an important target in interventions to help improve the overall physical and psychosocial health of individuals with SMI [7, 8].

### Physical activity and community-based group programmes

Physical activity is defined as any bodily movement produced by skeletal muscles that result in a substantial increase in energy expenditure [9]. In the context of this review, PA will encompass light, moderate and vigorous intensity activities and the specific terms ‘exercise’^1^ and ‘sport’^2^ will be used when appropriate. Community-based approaches to PA involve community members and leaders from various settings and organisations coming together to promote PA in an organised and integrated manner [10]. They can use limited resources to reach a large number of people and often result in greater improvements in outcomes and increased sustainability over time [10]. A community-based approach is appropriate for PA for SMI groups, whose health is influenced by complex individual-level and system-level factors [3] and who may have specific needs and barriers to PA participation [11–13].

### Health benefits of physical activity for people with SMI

Physical activity has many potential physical health benefits for people with SMI, including symptomatic improvement [14] and weight loss benefits [15]. Recent systematic reviews have concluded that there is, albeit mixed, evidence for its beneficial role on measurable health outcomes such as improved symptoms, quality of life, PA level, physical fitness, cardiometabolic risk factors, BMI and weight [11, 14, 16–18]. The mixed findings in the literature are likely to reflect the methodological quality of primary studies, which have been criticised for small sample sizes, lack of long-term follow-up and lack of theoretical framework [15, 16]. Despite potential health benefits of PA for individuals with SMI, it is known that individuals with SMI engage in less exercise, and significantly greater amounts of sedentary behaviour than the general population [7, 8]. Such findings suggest that attention should be given to *how* to promote the uptake of PA in this population to bring about health benefits.

### Initiation of physical activity by individuals with SMI

Health organisations such as the Department of Health in the United Kingdom (UK) recommend evidence-based PA interventions to improve the physical health of individuals with SMI [19]. Yet poor uptake of PA by this population remains a problem [7, 8]. Poor recruitment to PA interventions is often reported [20], with patients citing numerous physical health, psychological and socio-ecological barriers to PA [11–13]. Non-uptake of PA by people with SMI does not necessarily reflect a lack of motivation or intention to be active; Ussher et al. [12] found a high level of interest in PA among people with SMI, but low levels of activity. This implies that there might be a gap between intention and behaviour that can be addressed by exploring the experience of PA initiation in greater depth.

This review will focus on the initiation of PA, defined as the period in which people start being more physically active (also referred to as ‘adoption’ and ‘uptake’). The experience of initiation is likely to differ from the maintenance of PA. Soundy et al. [13] illustrated how autonomy and identity, experiences and needs of people with SMI change through phases of PA (pre-activity, within activity and post-activity). In the pre-activity phase, successful initiation depends on the provision of social support from introducing the patient to the idea of becoming more physically active through to introducing patients to the new activity environment [13]. The current review aims to further explore the experience of the initial uptake of PA by individuals with SMI to help inform how interventions can be designed to increase initiation.

### The role of social support

Social support and sustainable supportive relationships could help with PA initiation barriers in people with SMI who are known to experience social isolation and stigmatisation [13, 17, 21, 22]. Physical activity in group community-based settings has the additional benefits of social support that may encourage activity engagement and the creation of a social identity [13, 21]. It is also possible that the social orientation of the community-based activity may be more appealing for individuals with SMI than the activity itself [21]. There is a need to hear how people with SMI perceive and experience group community-based PA to better understand the key features of social support that may encourage uptake of PA by people with SMI.

### The experience of physical activity

Understanding how patients with SMI perceive their own illness and experience PA may be key to designing more acceptable and effective interventions that meet patients’ expectations and needs. Qualitative methodologies can capture accounts of patients’ experience of PA in the patients’ own words [23]. Patient experience represents a unique account of the journey experienced by people with health conditions that is challenging to measure objectively. In the context of this review, the patient experience will be used to explore how people with SMI experience the initiation of community-based group PA, including engagement, satisfaction, expectations, preferences, perceptions and participation [23].

In recent years, the patient experience of PA for people with SMI has been explored. Syntheses of the qualitative literature using a meta-ethnographic approach have provided a comprehensive overview of the available research and practical implications for practice [13, 24–26]. In 2012, Soundy and colleagues [13] explored how psychosocial factors impact on barriers and facilitators to activity among patients with SMI during the initiation and maintenance phase of participation. Soundy et al. identified social support as essential for initiation and called for further research to explore social support in greater detail, particularly how social support can be used to support PA uptake [13]. In 2014, Soundy et al. [25] explored the experiences of PA for individuals with schizophrenia from the perspective of patients and HCPs who worked with the individuals. Reviewers identified psychosocial benefits of PA including self-initiated changes in behaviour, improved autonomy, increased confidence and the important social value of PA. Soundy et al. [26] built upon these findings in 2015 and explored the broader psychosocial benefits of sports participation for individuals with SMI. They found that sport can benefit individuals with SMI by helping them to overcome the negative effects of social isolation [26].

The findings to date [13, 24–26] have been important for going beyond measurable outcomes of PA and demonstrating the broader psychological, social and environmental factors influencing the experience of PA and sport for individuals with SMI. The findings require further exploration beyond single conditions and specific contexts to elicit the unique features of social support within community-based PA programmes for people with SMI. Exploration of the patient experience of community-based group PA is needed to help understand how to overcome barriers and promote initiation in this population. This new understanding may be key in designing more acceptable and effective community-based group PA programmes that meet patients’ expectations and needs.

### Aim

This review will aim to address the research question: How do adults diagnosed with SMI experience the initiation of community-based group physical activity and key features of social support within these contexts?

### Objectives

This review will seek to:

- Systematically search and appraise the qualitative research on the experience of initiation of community-based group PA for people with SMI
- Synthesise findings from existing research regarding the experience of initiation of community-based group PA and key features of social support within these contexts for people with SMI
- Identify from participants’ experiences the active ingredients that could inform future PA programmes and optimise the uptake of community-based group PA among people with SMI

## Methods and design

The review has been registered in the International Prospective Register of Systematic Reviews (PROSPERO): CRD42017059948. This will use the meta-ethnographic approach by Noblit and Hare [27]. If published in time for our review, we will use new reporting guidelines for meta-ethnography developed from the eMERGe study [28]. This protocol has been developed in accordance with the Preferred Reporting Items for Systematic review and Meta-Analysis Protocols (PRISMA-P) (see Additional file 1) [29].

### Eligibility criteria

Studies will be selected according to the criteria outlined below.

### Study design

A study will be eligible for inclusion if it reports primary data and has used any type of qualitative methodology. This includes studies that have used mixed methods, provided it is possible to extract the findings from the qualitative strand of the study. Further, included studies must have been peer reviewed or be theses, or reported in grey literature. Review papers, editorial and opinion pieces will be excluded.

### Population

To be eligible for inclusion a study must focus on SMI in adult humans (≥18 years), defined as a primary diagnosis of schizophrenia, bipolar affective disorder, major depressive disorder, personality disorder, severe anxiety (including phobia and obsessive compulsive disorder), schizophreniform disorder or psychosis (including first episode psychosis). This includes a first diagnosis or relapse of a previously diagnosed condition. To be included, studies must demonstrate that >80% of the sample has a diagnosis of one or more of these conditions. Studies that include participants with a diagnosis of an eating disorder, mild or moderate depression, a learning disability, or drug and alcohol use disorder, and where the primary study findings are not attributable to participants with the included conditions will be excluded. Populations that include those with comorbidities, whether physical or mental, will be eligible for inclusion. Studies that report data on both adults and children will be eligible for inclusion provided it is possible to extract the adult data.

### Physical activity

Studies that report community-based group PA are eligible for inclusion in this review. This will include group-based light, moderate, or vigorous intensity PA in a community setting and also community-based group activities described as sport or exercise. For a multi-component intervention such as a healthy lifestyle programme to be eligible for inclusion, a study must report the PA as one of the main components of the intervention and the primary study findings must be attributable to the PA. Studies will not be excluded on the basis of the type, frequency or duration of the PA.

### Comparison

Any studies involving a community-based group PA intervention do not need to include a comparator condition to be eligible for inclusion in the review. Where a comparison is made, the comparator could be no activity or any other activity.

### Outcomes

Only studies that capture the experience of the initiation in community-based group PA for people living with SMI will be eligible for inclusion. Data must be gathered from the perspective of the participants. The outcomes reported (i.e., participants’ experiences of initiation of community-based group PA and key features of social support within these contexts) need to be based on having used qualitative data collection and analysis methods. Studies will be eligible for inclusion irrespective of whether follow-up outcomes were collected. When included, the length of follow-up will be reported.

### Setting

To be considered for inclusion in the review, the setting for PA must be a community-based group setting.

Resource constraints mean it will not be possible to consider non-English language papers for inclusion. Studies will not be excluded based on the date of publication as this area of research is not sensitive to a particular period of time.

### Information sources

The bibliographic databases searched will be: ASSIA (ProQuest interface), CINAHL (EBSCO interface), Cochrane Central Register of Controlled Trials (Wiley interface), EMBASE (Ovid interface via NICE), Health Technology Assessment Database (HTA) (Wiley interface), MEDLINE (EBSCO interface), PsycINFO (ProQuest interface), Sociological Abstracts (ProQuest interface), SPORTDiscus (EBSCO interface) and Web of Science (Thomson Reuters interface).

In addition, grey literature will be sought using NICE Evidence Search and searches of targeted organisations and web resources. All grey literature sources will be searched from their inception. ProQuest Dissertation and Thesis (ProQuest interface) will be searched for student theses.

Author, citation and reference searches will be undertaken on papers included in the review. Hand searches of the contents of key journals and conference proceedings will also be undertaken. Systematic review papers will not be considered for inclusion in the review, but will be used to cross-check that relevant studies have been identified. Finally, a bibliography of included studies will be shared among this review team and a stakeholder group with a view to identifying any relevant, missing studies.

### Search strategy

A draft search strategy has been devised and piloted by the review team and will also be reviewed by a stakeholder group.

The draft search strategy as written for MEDLINE (EBSCO interface) comprises three facets: ((“mental illness” OR “mentally ill” OR “mental ill health” OR depression OR “depressive disorder*” OR bipolar OR schizophre* OR psychosis OR psychoses OR OR “mood disorder*” OR “dissociative disorder*” OR “personality disorder*” OR “obsessive compulsive” OR anxiety OR phobi* OR mentally ill persons/ OR mental disorders/ OR exp.anxiety disorders/ OR exp.bipolar and related disorders/ OR exp.dissociative disorders/ OR exp.mood disorders/ OR exp.personality disorders/ OR exp.schizophrenia spectrum and other psychotic disorders/) AND (physical* N3 activ* OR exercis* OR “motor activity” OR sport* OR “physical therap*” OR “physical training” OR physical* N3 fit* OR motor activity/ OR exp.exercise/ OR exp.sports/ OR physical fitness/) AND (experience* OR perspective* OR story OR stories OR narrative* OR ethnograph* OR qualitative OR narration/ OR exp.personal narratives/ OR exp.anthropology, cultural/ OR exp.qualitative research/)).

All search terms will be looked for in the title and abstract fields, and controlled vocabulary terms will be used where available. The Boolean operators AND and OR will be used, alongside truncation (*), phrase searching (“”) and proximity operators (N). When available as a search limiter, the results will be filtered to only include English language publications. No date limits will be applied.

Once the final search strategy has been agreed, the search syntax, and if available the controlled vocabulary terms, will be adapted for use on the other information resources to be searched. The literature searches will be updated towards the end of the review process to ensure that no newly published studies are missed.

### Data management

The bibliographic management tool EndNote will be used to organise the literature in this review. Duplicate results will be removed within EndNote and both the number of results before and after duplication will be recorded. Microsoft Excel 2010 will be used to support the literature selection and data extraction processes. The selection and data extraction processes will be preceded by the review team having completed independent calibration exercises to ensure familiarity with the IT tools being used and to refine and maximise consistency in approach.

### Selection process

The titles and abstracts of all search results will be examined for relevancy by one reviewer, and 10% of the records excluded at title and abstract will be checked by a second reviewer. The relevancy of a study will be judged by the inclusion/exclusion information detailed in the eligibility criteria section and in the order: population, setting, physical activity, outcomes and study design. The full-text of studies will be obtained when the inclusion criteria are met or when it is impossible to determine the eligibility of a study using only the title and abstract.

Study selection based on the full-text will use the same inclusion/exclusion criteria and approach to judging the relevancy of a study. It will be decided by two independent reviewers, with discrepancies resolved by discussion. If necessary, a third team member will be involved. During the full-text screening process, the reason for excluding a study will be recorded. The corresponding author of a study will be contacted via email if further study details are required. Two attempts will be made to contact the corresponding author. When the full-text of a study is not available via the review team’s libraries, it will be requested from the corresponding author/s or from the British Library.

None of the reviewers involved in the study selection process will be blinded to information about the authors, or the journal title. Inter-rater agreement at both stages of the selection process will be calculated.

### Data collection process

Data will be extracted from all included studies by one reviewer using a standardised data extraction form piloted on at least two studies. Data relating to findings will be extracted verbatim from primary studies, and quotations will be recorded where appropriate. The data extraction process will also be used to identify linked studies or studies using the same data. These studies will be identified by checking information such as the authors, the dates a study was undertaken, and the sample size and characteristics.

All extractions will be checked thoroughly by a second reviewer who will also have independently completed a pilot data extraction exercise on at least two studies. Discrepancies will be resolved by discussion, and with reference to a third team member if necessary. The data extracted from each study will then be transposed from the standardised forms into a tabular presentation which will provide a summary of the data items and outcomes, with a different row being used for each study.

### Data items

Desired data items not reported in a study will be recorded as not available. If unavailable data is considered to be of a high priority then two attempts will be made to contact the corresponding author for clarification. The bibliographic details of each study will be extracted, alongside information about sources of financial and non-financial support when reported. The details of potentially relevant references will also be extracted. Study information, including details of the study location, research question and/or aim and objectives, data collection methods, validation and recording, and the data analysis process will be extracted. The participant information extracted will be selected demographic information (age, gender, ethnicity, employment status, other health conditions), SMI diagnosis, length of diagnosis, if the occurrence of the SMI is first episode or relapse, information on the resultant disability and the severity of the SMI, as well as information about contact with mental health services and care workers more generally, and details of recruitment and sampling. Physical activity information extracted will be: type of activity, if the activity is self-initiated or referred, the type of environment in which the activity is undertaken, how the intervention is integrated within the wider community, details of the intensity, frequency and duration/distance, if the activity includes elements of a behavioural change intervention, as well as details of interactions with mental health services and care workers as a result of the PA intervention.

Data outcomes to be extracted must fulfil the following criteria: (1) determined by the author of the study to be at the least partially attributable to the PA; and (2) relate to the experience of initiating PA from the perspective of the participants. Other findings included in a study, but not fulfilling the aforementioned criteria will not be extracted. Data on findings will be extracted thematically and include sub-themes where required. The method of reporting findings will be recorded (e.g., as verbatim quotes from participants or as statements from the author). The conclusions of the primary author/s will also be extracted. In addition, reviewers will record any of their own comments, such as any limitations of the study or paper.

### Quality appraisal of individual studies

Two reviewers will critically appraise each included study to explore the quality of the methods and reporting. Whilst critical appraisal is incumbent to a quantitative systematic review; the inclusion of such a process in qualitative reviews is supported [30–32]. Critical appraisal can identify weaknesses in a study that may impact on the validity of study findings, and then collectively the findings reported in the review.

The quality of studies will be assessed using the Critical Appraisal Skills Programme (CASP) Qualitative Checklist [33]; a validated instrument that has been used in previous meta-ethnographies [30, 34, 35]. Use of this checklist will guide the process and offer transparency as the reviewers will need to consider an identical set of structured questions. Previous meta-ethnographic studies that have used the CASP quality assessment tool (e.g., Atkins et al. [35]) have found it useful to appraise the quality of the written report and found several methodological problems that included failure to report the qualitative approach used, the theoretical orientation of the researchers, the theoretical framework used, and the approach to analysis.

Critical appraisal will be performed by one reviewer and independently double-checked by a second reviewer. Discrepancies in the assessments made by the reviewers will be resolved by discussion, with involvement of a third reviewer if necessary. All reviewers will independently pilot the quality assessment tool on at least two studies. None of the reviewers involved in the quality assessment process will be blinded to information about the authors, or the journal title. Inter-rater agreement will be calculated. Studies will not be excluded on the basis of quality appraisal outcomes, an approach aligned with Thomas and Harden [36] who assert that ‘there is little empirical evidence on which to base decisions for excluding studies based on quality assessment’ (page number unknown).

The quality assessment across all studies and for all ten CASP Qualitative Checklist questions will be transposed into a tabular form. In addition, a descriptive summary of the methodological quality of each study will be included as a column in the tabular presentation of the data items and outcomes extracted from each study.

### Data synthesis

**Fig. 1:**
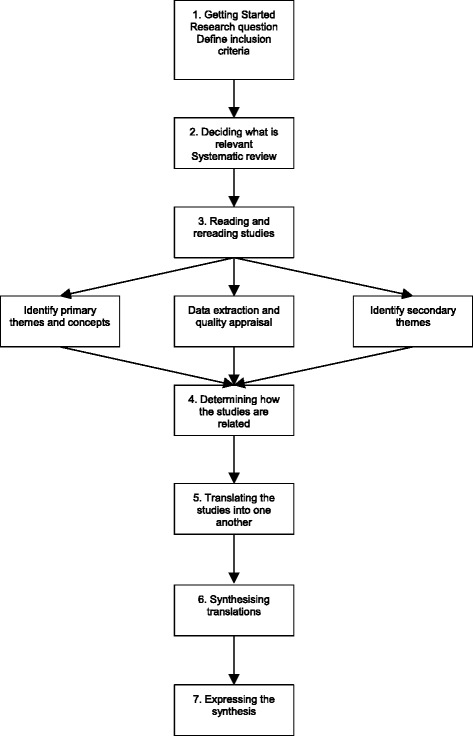
Meta-ethnographic approach according to Noblit and Hare [27] adapted from Monforte-Royo et al. [40]

The tabular presentation summarising each study (data items and outcomes, and methodological quality) will help to identify the patterns and relationships within and between studies, thus facilitating the data synthesis process. The seven-stage meta-ethnographic approach developed by Noblit and Hare [27] will be used to synthesise the data from the studies included (see Fig. 1). Meta-ethnography is an interpretive approach to synthesis, rather than the aggregative approach of traditional meta-synthesis of quantitative research, making it appropriate for this review. Rather than rigidly adhering to the steps in sequential order, we will use some flexibility to allow for ongoing and simultaneous synthesis within an iterative and organic approach.

Steps 1–3 will involve the systematic literature search, reading of the articles, quality appraisal and data extraction outlined above. If during these steps it becomes clear that a large number of papers meet the inclusion criteria, through discussion, the authors will develop a strategy to select a sample of studies based on factors such as study design, population, intervention and outcomes. Step 4 will involve determining how the studies are related. Key themes and concepts identified in individual studies will be examined in relation to other studies. Step 5 will involve translating the findings from primary studies and exploring how these relate to other studies using Noblit and Hare’s meta-ethnographic techniques of reciprocal translations, refutational synthesis and lines of argument synthesis. This will involve summarising the themes and concepts extracted from primary studies and comparing them across studies in attempt to ‘match’ themes if they are deemed sufficiently similar (reciprocal translations). Primary articles will be read and re-read and the findings constantly compared to ensure the translations are grounded in the original studies. During this phase, any contradictions between studies will be explored and explained (refutational synthesis). In Step 6, themes can be reconceptualised and a new interpretation generated to form a line of argument, aiming for a final synthesis that is greater than the sum of its parts (line of argument synthesis). This approach allows the language used in primary studies to be maintained while creating new metaphors within the current synthesis [13]. Emergent themes will be discussed with the review team. Step 7 will involve presenting the results.

### Meta-bias

The possibility of publication bias will be minimised through the planned inclusion of grey literature searches and searches for student theses. However, publication bias achieved as a result of non-publication remains possible. Any selective reporting of outcomes within studies included in this review will be recorded. Selective reporting will be checked for by comparing the outcomes reported in a study’s methods section with those reported in the results section.

### Confidence in cumulative evidence

The Confidence in the Evidence from Reviews of Qualitative research (CERQual) framework [37] will be used as a tool to help evaluate the strength of the review findings. In doing so it will provide readers of the review with a level of confidence in the different findings presented. The quality of the findings will be considered across the CERQual components as follows: methodological limitations; relevance; coherence; and adequacy of data. Finally, for each finding, a descriptive summary of the strength of the evidence will be produced.

### Patient and public involvement

To inform the review, the involvement of local SMI patient groups will be sought. The group will: i) advise on study selection wherever necessary; ii) sense check themes and findings identified during synthesis; and iii) assist with the drafting of the plain English summary for the dissemination of findings.

## Discussion

Active lifestyles among people living with a SMI may help relieve the severe and debilitating symptoms that can negatively influence their overall health and quality of life leading to premature mortality, morbidity and obesity. The proposed review of the qualitative literature exploring the experience of initiation of community-based group PA for people living with a SMI will deepen the understanding of how PA initiation is experienced by this population in this context. With this deeper understanding, researchers, practitioners and policy makers will be in a better position to understand patients’ needs and expectations, implement interventions and promote the initiation of active lifestyles in this population.

## Additional file

Additional file 1: Quirk et al. PRISMA-P + checklist.docx - Completed PRISMA Checklist. (DOCX 36 kb)

## Abbreviations

CASP: Critical Appraisal Skills Programme
CERQual: Confidence in the Evidence from Reviews of Qualitative research
PA: Physical activity
PRISMA-P: Preferred Reporting Items for Systematic reviews and Meta-Analysis Protocols
PROSPERO: International Prospective Register of Systematic Reviews
SMI: Serious mental illness

## Footnotes

1 Exercise is a form of PA that is “planned, structured, repetitive, and purposive in the sense that improvement or maintenance of one or more components of physical fitness is an objective” ([38], p. 128).

2 Sport will be defined as games with rules that require motor actions and that can be organised or unorganised and conducted at competition or leisure level, such as tennis ([39], 1992).
